# The kinetic behavior of matrine in pig intestinal lumen after oral administration and its physiologically based pharmacokinetic modeling

**DOI:** 10.3389/fvets.2025.1620161

**Published:** 2025-10-14

**Authors:** Bo Yang, YiWei Jia, FuHao Wang, XiaoLing Lv, SuYang Ma, YaXin Tan, WanXin Zhang, Dan Wan, Rui Li, DanNa Zhou, DaoJin Yu

**Affiliations:** ^1^University Key Laboratory for Integrated Chinese Traditional and Western Veterinary Medicine and Animal Healthcare in Fujian Province, Fujian Key Laboratory of Traditional Chinese Veterinary Medicine and Animal Health, College of Animal Sciences, Fujian Agriculture and Forestry University, Fuzhou, China; ^2^Fujian Sunner Development Co. Ltd, Nanping, China; ^3^Institute of Subtropical Agriculture, Chinese Academy of Sciences, Changsha, China; ^4^Key Laboratory of Prevention and Control Agents for Animal Bacteriosis (Ministry of Agriculture and Rural Affairs), Hubei Provincial Key Laboratory of Animal Pathogenic Microbiology, Institute of Animal Husbandry and Veterinary, Hubei Academy of Agricultural Sciences, Wuhan, China

**Keywords:** physiologically based pharmacokinetic model, matrine, pig intestinal lumen, pharmacodynamic evaluation, liquid chromatography tandem mass spectrometry

## Abstract

**Introduction:**

Matrine (MT) has been found to restore the susceptibility of *Escherichia coli* to a variety of antibiotics in vitro. Nevertheless, the absence of pharmacokinetic data makes it uncertain whether MT exhibits efficacy *in vivo*. The study aimed to investigate the kinetic behavior of MT in pig intestinal lumen, the primary site for the colonization of enterotoxigenic *Escherichia coli*, and to develop a minimal physiologically based pharmacokinetic (PBPK) model for MT in pig intestinal lumen.

**Methods:**

Two animal experiments were carried out for these purposes. In experiment 1, 12 pigs were implanted with a sterile T-cannula, and then were given a single oral dose of MT or MT-Amoxicillin (AMO) combination at 40 or 70 mg/kg. In experiment 2, 25 pigs were administered with MT at 50 mg/kg/d by oral gavage for 5 d. Intestinal contents were collected at predetermined times and analysed by liquid chromatography tandem mass spectrometry (LC–MS/MS) method. The concentration-time data were analysed by non-compartmental method. Subsequently, a four-compartment PBPK model was developed and validated.

**Results:**

After oral administrations, the MT concentrations in pig intestinal lumen increased rapidly and reached their peaks within 2 h, then decreased in a two-phase decay pattern. The co-administered AMO did not alter the kinetic behavior of MT in pig intestinal lumen. The PBPK model gave an accurate prediction of MT concentrations in pig intestinal lumen at most time points.

**Discussion:**

A dosage regimen of 70 mg/kg every 8 h was recommended to ensure a sufficient drug exposure.

## 1 Introduction

Porcine colibacillosis is an infectious bacterial disease commonly caused by enterotoxigenic *Escherichia coli*. It is usually clinically manifested as watery diarrhea, dehydration, depression, and inappetence, and has a widespread and detrimental impact on pig health and economics of pig industry (1). Antibiotics such as *β*-lactams, aminoglycosides, and fluoroquinolones are used for the treatment of porcine colibacillosis. Unfortunately, increasing antibiotic resistance has been observed in enterotoxigenic *Escherichia coli* (1, 2), making these drugs less effective. Some phytochemicals exhibit unique antibacterial activities, and their use alone or in combination with existing antibiotics may become a promising anti-infective treatment strategy (3). Matrine (MT) is a quinolizidine alkaloid isolated from *Sophora flavescens Ait* and *Sophora alopecuroides* (4). It is occasionally used to kill the ectoparasites (such as sarcoptic mite) of some animals. Recently, MT has been found to restore the susceptibility of *Escherichia coli* (*E. coli*) to a variety of antibiotics *in vitro* by inhibiting the AcrAB-TolC efflux pump (5, 6). Another study revealed the inhibitory effect of MT on biofilm formation of antimicrobial-resistant *Escherichia coli*, which helped to increase the susceptibility of these strains to antibiotics (7). Such multidrug resistance reversal activity makes MT a potential therapeutic or prophylactic drug for porcine colibacillosis.

However, it remains unclear whether MT is effective *in vivo*. An important reason is the lack of pharmacokinetic (PK) data, since the time course of drug concentration in the body, especially at the site of infection, is thought to be closely related to the therapeutic efficacy (8). For porcine colibacillosis, the small intestinal epithelial cells are the main site of enterotoxigenic *E. coli* colonization (9). This makes the time course of MT concentrations in pig intestinal contents far more critical than conventional plasma PK profiles for accurately evaluating its *in vivo* efficacy. To our knowledge, the plasma PK profiles of MT have been described in humans (10), dogs (11), rabbits (12), and rats (13–17), but no data are available on the PK of MT in either plasma or, more importantly, the intestinal lumen of pigs.

Physiologically based pharmacokinetic (PBPK) models are powerful tools for pharmacodynamic evaluation and dosage regimen design in veterinary practice. Compared with traditional compartment models, they can quantitatively predict the time course of drug concentration at specific sites of interest (such as plasma, urine, organs, or sites of infection), and extrapolate to different doses, formulations, or species. Gao and Law (15) reported an eleven-compartment, flow-limited PBPK model for predicting the disposition of MT in rat. However, there is no PBPK model for predicting the kinetic behavior of MT in pig intestinal lumen available.

Based on these considerations, we investigated the kinetic behavior of MT in pig intestinal lumen after oral administration of MT alone and in combination with amoxicillin (AMO), and developed a minimal PBPK model to evaluate the *in vivo* efficacy of MT and to recommend an appropriate dosage regimen.

## 2 Materials and methods

### 2.1 Chemicals and reagents

The analytical standard of MT (CAS no. 519-02-8, purity > 98.0%) was purchased from Shanghai yuanye Biotechnology Co., Ltd. (Shanghai, China). LC/MS grade acetonitrile and formic acid were purchased from Merck KGaA (Darmstadt, Germany). LC/MS grade ammonium acetate was purchased from Sigma-Aldrich (St Louis, MO, USA). Analytical grade methanol, ethyl acetate, and trichloroacetic acid were purchased from Sinopharm Chemical Reagent (Shanghai, China). Ultrapure water (18.3 MΩ*cm) was used throughout the study. A stock solution of MT was prepared at 1000 μg/mL in acetonitrile and stored at −20°C. Working standards were prepared by diluting the stock solution in acetonitrile, and were stable at 4°C for at least 1 week. AMO soluble powder (30%) was purchased from Wuhan Hvsen Biotechnology Co., Ltd. (Wuhan, Hubei Province, China).

### 2.2 Animals

Twelve crossbred (landrace × large white × duroc) pigs, with body weights (BW) ranging from 24.3 to 31.1 kg were purchased from Tangrenshen Group Co., Ltd. (Zhuzhou, China). Each pig was housed separately in a stainless steel metabolic crate (1.4 m × 0.7 m × 0.5 m). These crates were placed in a well-ventilated room with temperature of 25.8 ± 3.2°C and humidity of 76.5–87.1%. Another 25 crossbred (landrace × large white) pigs, with BW ranging from 9.8 to 10.3 kg were purchased from Fujian Minlv Three Dimensional Agricultural Comprehensive Development Co., Ltd. (Ningde, China). They were housed in six 5 m × 3 m pens, in which the temperature and humidity were maintained at 21.4 ± 6.2°C and 72.2–88.4%, respectively, and good ventilation was ensured. All animals were determined to be clinically healthy prior to study inclusion, based on normal results from physical and neurologic examinations, hematologic evaluation, and urinalysis—along with targeted assessment of intestinal health, including observation of normal appetite, fecal consistency, and absence of gastrointestinal signs (e.g., diarrhea, abdominal distension). During the experiment, the pigs were fed with drug-free feed and water. A 7-d adaptation period was allowed prior to the experiment. The animal experiments were approved by the Research Ethics Committee of the College of Animal Science, Fujian Agriculture and Forestry University (No. PZCASFAFU21031).

### 2.3 Experimental design

Two animal experiments were carried out in this study. Experiment 1, which aimed to elucidate the kinetic behavior of MT in the pig intestinal lumen, utilized 12 crossbred (Landrace × Large White × Duroc) pigs. The sample size (6 individuals per group) for experiment 1 was determined both based on the experimental objective and in accordance with Announcement no. 1247 of the Ministry of Agriculture of the People’s Republic of China (18). Experiment 2, primarily designed to generate data for evaluating the performance of the developed PBPK model, employed 25 crossbred (Landrace × Large white) pigs. For experiment 2, the sample size (5 individuals per time point) was determined specifically with reference to its experimental purpose.

In experiment 1, the pigs were randomly assigned to two groups with 6 replicates per group, namely group A and group B. Each pig was surgically implanted with a sterile T-cannula into the terminal ileum approximately 15 cm cranial to the ileocecal valve. The T-cannula was custom-fabricated from rigid silicone, with its main structure consisting of a grooved section and a cylindrical tube. Specifically, the cylindrical tube measured 25 mm in length, 18 mm in inner diameter, and 31 mm in wall thickness; it was externally threaded with a pitch of 1 mm, which allowed for secure fitting with a matching nut to ensure post-implantation stability. The implantation of T-cannula was performed according to a standard procedure (19). After the implantation procedure, the pigs were subjected to a 7-day post-operative recovery period. During this recovery period, each pig received daily intramuscular injections of sodium penicillin at a dosage of 20,000 U/kg to prevent post-surgical infection. Subsequently, an additional one-week acclimation period was provided to ensure the animals reached a state of physiological stability before proceeding with subsequent experiments. The pigs in group A received a single oral dose of MT at 40 mg/kg, while the pigs in group B received a single dose of MT at 40 mg/kg and AMO at 40 mg/kg. Intestinal contents (approximately 2 g) were collected via the T-cannula at 0.25, 0.5, 1, 2, 3, 4, 5, 8, 12, 16, 24, 36, 48, 72, and 120 h post-dosing, and stored at −20°C until analysis. After a 2-week washout period, the protocol was repeated with a higher dose (70 mg/kg).

In experiment 2, the pigs were administered MT at 50 mg/kg/d by oral gavage for 5 d, consecutively. Five pigs were randomly sacrificed at 0.5, 1, 3, 6, and 12 d after the last administration. The intestinal contents from each pig were separately collected in marked containers, and stored at −20°C until analysis.

### 2.4 Analysis of intestinal content samples

Pig intestinal contents were obtained from two sources: the local slaughterhouses and the animal experiments (experiments 1 and 2). Samples from the local slaughterhouses were confirmed to be negative for MT and then used for the validation of the analytical method. Samples from subsequent animal experiments (the incurred samples) were used for the elucidation of the kinetic behavior of MT in pig intestinal lumen and the development of PBPK model. Subsamples of 1 g were weighed in a 50-mL polypropylene centrifuge tubes and extracted by vortexing with 12 mL of acetonitrile for 5 min. After centrifugation at 6000 rpm for 5 min, the supernatant was transferred and evaporated to dryness at 45 °C, under nitrogen flow. The solid residue was dissolved with 2 mL of water. Then, 4 mL of ethyl acetate was added. The mixtures were vortexed for 30 s and centrifuged at 6000 rpm for 5 min. The ethyl acetate extract was discarded. The remaining aqueous phase was diluted to 6 mL with 3% trichloroacetic acid solution and loaded onto a X-Peony mixed-mode cation exchanger (MCX) solid phase extraction (SPE) cartridge (300 mg, 6 mL) (Guangzhou Finnegan Instrument Co., Ltd., Guangzhou, China) previously conditioned with 3 mL of methanol, 3 mL of water, and 3 mL of 3% trichloroacetic acid solution. The cartridge was washed with 3 mL of 0.25% ammonium hydroxide solution, 5 mL of water, and 5 mL of methanol, airdried and eluted with 6 mL of ammonium hydroxide-methanol mixture (10:90, v/v). The elution collected was evaporated to dryness under nitrogen stream. The residue was reconstituted in 1 mL of water and filtered through a 0.22-μm PTFE filter before injecting it into the LC–MS/MS system.

Analyses were performed on an Agilent 6,460 Triple Quadrupole LC–MS system (Agilent Technologies Inc., Santa Clara, CA, USA). The chromatographic separation was accomplished on a ChromCore C18 column (2.1 × 100 mm, 3 μm; NanoChrom Technologies Co., Ltd., Suzhou, China) at 35 °C. The mobile phase A was 1% formic acid solvent (containing 5 mmol/L ammonium acetate), while the mobile phase B was acetonitrile. The gradient was as follows: 0–0.2 min, A/B (80/20); 0.2–2 min, A/B (50/50); 2–2.5 min, A/B (80/20); 2.5–3 min, A/B (80/20). The flow rate was 0.2 mL/min, and the injection volume was set at 2 μL. MT was ionized in positive electrospray ionization (ESI+) mode and detected in multiple reaction monitoring (MRM). The qualitative and quantification ions were *m/z* 249.1 → 148.0, *m/z* 249.1 → 176.0, and m/z 249.1 → 148.0, respectively. Specific mass spectrometer (MS) parameters were set as follows: capillary voltage of 3.0 kV, source temperature of 350°C, desolvation gas (nitrogen) flow of 720 L/h, and desolvation temperature of 350°C. Other parameters were shown in Supplementary Table S1.

The method was validated according to guidelines published by the United States Food and Drug Administration (20). Validation parameters included matrix effect, linearity, specificity, precision, accuracy, sensitivity, and stability. The matrix effect was determined as described previously (21). A matrix-matched calibration curve was used to calculate the MT concentration in pig intestinal contents. The curve was built by spiking blank sample extracts with known concentrations of MT (1–100 μg/L). The linearity was represented by the correlation coefficient. The quality control (QC) samples with final concentrations of 2, 20, and 100 μg/kg were prepared by adding appropriate volumes of working standards to blank samples. Twenty blank samples from different sources and 18 QC samples were analysed as described above. Specificity was evaluated by comparing the MRM chromatograms of blank samples with those of QC samples. The QC samples were also used to evaluate accuracy, precision, sensitivity, and stability. The relative standard deviation (RSD) and relative error (RE) were calculated by analyzing six replicate QC samples on the same day and on three consecutive days. The lower limit of quantification (LLOQ) was the lowest concentration of MT that could be quantitatively determined (RSD and RE ≤ 20%). Stability was also evaluated under various conditions (1 week at −20 °C, 24 h at room temperature (26 °C), and 3 freeze–thaw cycles).

### 2.5 Pharmacokinetic analysis

The MT concentration in intestinal contents, PK parameters, and all other data were presented as means ± standard deviation (SD). The PK parameters for each pig were determined with WinNonlin version 5.2.1 (Pharsight Co., Mountain View, CA, USA) using non-compartmental analysis. The maximum concentration (C_max_) and time to maximum concentration (T_max_) were derived directly from the concentration–time curve. The area under the curve from time 0 to 120 h (AUC_0 → 120 h_) was calculated using the linear trapezoidal rule. The terminal elimination rate constant (λ_z_) was estimated via linear regression of the terminal log-linear phase of the concentration-time curve, and the terminal elimination half-life (t_1/2_) was subsequently calculated as 0.693/λ_z_. The mean residence time (MRT), clearance per kilogram of body weight (CL/F), and apparent volume of distribution per kilogram of body weight (V/F) were calculated using standard non-compartmental formulas based on dose, AUC_0 → ∞_, and AUMC_0 → ∞_ (area under the first moment curve from time zero to infinity). The differences in PK parameters between the groups A and B were determined by a two-tailed t-test using SPSS version 21 (IBM Co., Armonk, NY, USA), and a *p* < 0.05 was considered significant.

### 2.6 Development of PBPK model

Prior to model development, the following assumptions were proposed with regard to the disposition of MT in pigs. It was hypothesized that (i) all rate processes involved in the model were kinetically first-order; (ii) all compartments in the model were well stirred, and the dispositions of MT in these compartments could be described by a flow-limited approach; (iii) the concentration of MT in pig intestinal lumen was equal to the corresponding value in pig intestinal contents; (iv) biliary excretion occurred when MT molecules passed through the liver.

**Figure 1:**
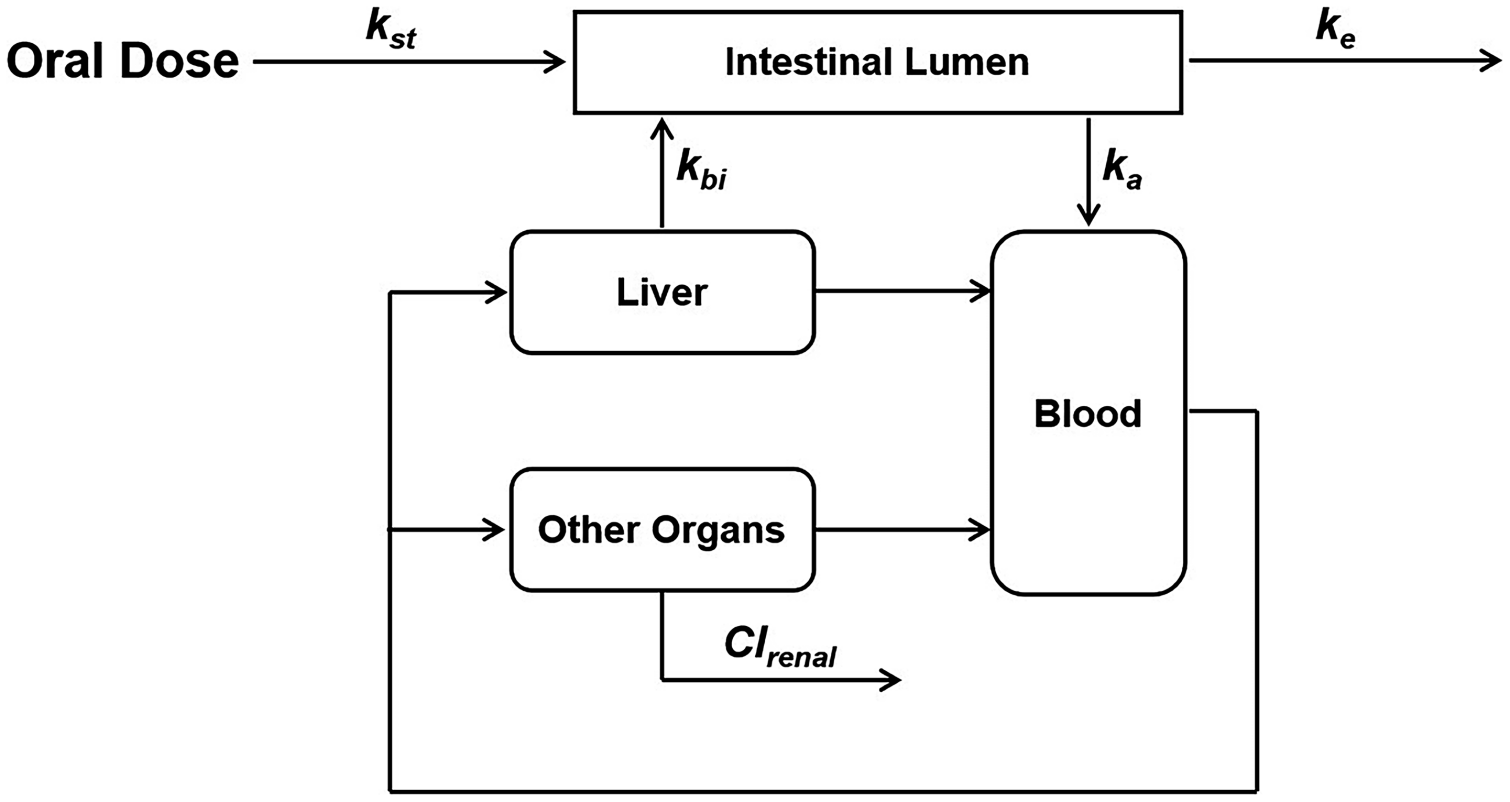
Schematic structure of the physiologically based pharmacokinetic model. k_st_ is the rate of gastric emptying, k_a_ is the absorption rate constant, k_e_ is the fecal excretion rate constant, k_bi_ is the biliary excretion rate constant, Cl_renal_ is the renal clearance.

A minimal flow-limited PBPK model was developed based on these hypotheses (Figure 1). The model structure included only the core compartments such as intestinal lumen, liver, and blood, while the remaining part of the body was lumped into a compartment named “other organs.” There was no blood supply to the intestinal lumen. The change in MT concentration in the intestinal lumen depended on gastric emptying, intestinal absorption, biliary excretion, and fecal excretion. The tissue compartments (liver and other organs) were connected by the blood flow. Renal excretion was incorporated in other organs, and was the major elimination pathway for MT to leave the body. The rate of change in MT concentration in each compartment was described by mass-balance differential equations (Table 1). Physiological parameters such as the rate of gastric emptying (k_st_), organ volume (Vc_i_), cardiac output (Qcar), and organ blood flow (Qc_i_) were obtained from the literature (22–24). Compound-specific parameters such as the bioavailability (F), absorption rate constant (k_a_), fecal excretion rate constant (k_e_), tissue-to-blood partition coefficient of liver (P_l_), and renal clearance (Cl_renal_) were obtained from previously published studies (14, 15). The plasma protein binding (Pb) and biliary excretion rate constant (k_bi_) were estimated from our intestinal PK studies by visually comparing simulations to appropriate data. Adjustments were made on all the above parameters to achieve the best fit to the experimental PK data using a maximum likelihood estimation algorithm (Nelder–Mead). The concentration-time data after oral administration of 40 mg/kg of MT alone was used for this purpose. Prior to model parameterization, a sensitivity analysis was conducted following the procedures described by Campbell (25), aiming to evaluate the effect of each parameter on model predictions. Each parameter was changed by 0.1% and then the rate of change in model prediction was computed. The sensitivity coefficients were calculated by the central difference formula using AcslXtreme OptStat module (Aegis Technologies, Huntsville, AL). Normalization relative to response variables involves dividing the computed sensitivity coefficient at each time slice by the response variable value at that time, while parameter normalization entails multiplying the computed sensitivity coefficient at each time slice by the parameter value. The sensitive parameters with the most significant impact on model were identified based on the absolute value of the normalized sensitivity coefficient (NSC) exceeding 0.1. Subsequently, the parameter optimization of these sensitive parameters was conducted in batches, with all other parameters held at their fixed values throughout the process. Importantly, strict limits were set for parameter adjustments to ensure that all optimized values remained within biologically plausible and experimentally acceptable ranges. All mathematical operations were performed using acslXtreme version 2.5.0.6 (Aegis Technologies, Huntsville, AL).

**Table 1:** Mass-balance differential equations to describe the rate of change of MT in compartments.

Compartment	Mass-balance differential equation
Intestinal lumen	$V_{\mathrm{int}}•\frac{{\mathrm{dC}}_{\mathrm{int}}}{\mathrm{dt}}=\text{DOSE}•k_{\mathrm{st}}+{\text{amount}}_{l}•k_{\mathrm{bi}}-{\text{amount}}_{\mathrm{int}}•k_{a}-{\text{amount}}_{\mathrm{int}}•k_{e}$
Liver	$V_{l}•\frac{{\mathrm{dC}}_{l}}{\mathrm{dt}}=Q_{l}•(C_{a}-\frac{C_{l}}{P_{l}})-{\text{amount}}_{l}•k_{\mathrm{bi}}$
Other organs	$V_{\mathrm{ot}}•\frac{{\mathrm{dC}}_{\mathrm{ot}}}{\mathrm{dt}}=Q_{\mathrm{ot}}•(C_{a}-\frac{C_{\mathrm{ot}}}{P_{\mathrm{ot}}})-C_{a}•{\mathrm{Cl}}_{\text{renal}}$
Blood	$V_{b}•\frac{{\mathrm{dC}}_{b}}{\mathrm{dt}}={\text{amount}}_{\mathrm{int}}•k_{a}+Q_{l}•(\frac{C_{l}}{P_{l}})+Q_{\mathrm{ot}}•(\frac{C_{\mathrm{ot}}}{P_{\mathrm{ot}}})-Q_{\mathrm{tot}}•C_{a}•(1-\mathrm{Pb})$

C_int_, C_l_, C_ot_, and C_b_: the MT concentration in intestinal lumen, liver, other organs, and blood, respectively; dC_int_, dC_l_, dC_ot_, and dC_b_: the change in MT concentration in intestinal lumen, liver, other organs, and blood, respectively; dt: the change in time; V_int_, V_l_, V_ot_, and V_b_: the volume in intestinal lumen, liver, other organs, and blood, respectively; Q_l_ and Q_ot_: the blood flow to liver and other organs, respectively; Q_tot_: the cardiac output; amount_int_ and amount_l_: the amount of MT in intestinal lumen and liver, respectively; DOSE: the oral dose; k_st_. k_e_, k_a_, and k_bi_: the rate of gastric emptying, the fecal excretion rate constant, the absorption rate constant, and the biliary excretion rate constant, respectively; Cl_renal_: the renal clearance of MT; P_l_ and P_ot_: the tissue-to-plasma partition coefficient of MT in liver and other organs, respectively; Pb: the plasma protein binding of MT.

The performance of our model was evaluated by visual inspection of the overlays of predicted and observed concentration-time profiles. Data sets required for performance evaluation were from our intestinal PK studies. It should be pointed out that there were four different sets of concentration-time data produced by experiment 1, and these data sets reflected the kinetic behavior of MT in pig intestinal lumen at various dosage regimens (MT alone, 40 mg/kg; MT alone, 70 mg/kg; MT in combination with AMO, 40 mg/kg; MT in combination with AMO, 70 mg/kg). Three of the above four data sets, coupled with another data set provided by experiment 2 were used for performance evaluation. All data sets used for model development were summarized in Table 2. Linear regression analysis and Pearson correlation test were carried out to evaluate the correlations between predicted and observed values. One-sample t test was carried out to examine the differences between the predicted PK parameters and the corresponding values derived from experiment 1. Linear regression analysis was performed using Microsoft Office Excel version 2020 (Microsoft Coperation, Redmond, WA, USA), and all statistical analyses were performed using IBM SPSS Statistics version 20.0 (IBM Corp., Armonk, NY, USA).

**Table 2:** Description of the data sets used for model development.

Dosage regimens	Route of administration	Number of animals	Bodyweight	Data source	Use of data sets
MT alone, 40 mg/kg	Feed medication	6	24.3–31.1 kg	Experiment 1	Model parameterization
MT in combination with AMO, 40 mg/kg	Feed medication	6	24.3–31.1 kg	Experiment 1	Performance evaluation of the PBPK model
MT alone, 70 mg/kg	Feed medication	6	24.3–31.1 kg	Experiment 1	Performance evaluation of the PBPK model
MT in combination with AMO, 70 mg/kg	Feed medication	6	24.3–31.1 kg	Experiment 1	Performance evaluation of the PBPK model
MT alone, 50 mg/kg once daily for 5 d	Oral gavage	25	9.8–10.3 kg	Experiment 2	Performance evaluation of the PBPK model

## 3 Results

### 3.1 Validation of the analytical method

**Figure 2:**
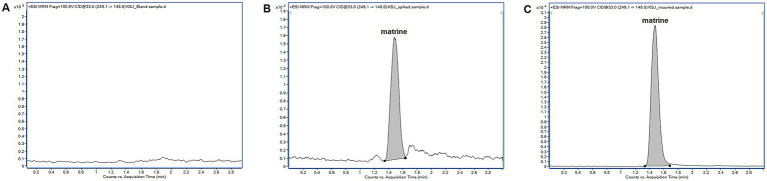
Representative multiple reaction monitoring chromatograms of matrine in pig intestinal contents. **(A)** blank sample; **(B)** blank sample spiked with MT at 20 μg/kg; **(C)** incurred sample collected from a pig 1 h after a single oral administration of matrine (40 mg/kg). The incurred sample was diluted with blank sample extracts prior to liquid chromatography tandem mass spectrometry analysis.

Representative MRM chromatograms obtained from blank sample, blank sample spiked with MT, and an incurred sample collected after administration were presented in Figure 2. The retention times of MT was approximately 1.478 min. There were no interfering peaks from endogenous compounds observed at the retention time of MT. The peak area ratios of MT at the concentrations of 2, 20, 100 μg/kg were 3.64 ± 0.26%, 3.67 ± 0.25% and 4.44 ± 0.23%, respectively. There were significant matrix suppression effects in pig intestinal contents. The matrix-matched calibration curve showed good linearity (y = 602.37x–96.672, r = 0.9997) over the concentration range from 1 to 100 μg/L. The accuracy (measured as RE) ranged from −9.87 to 4.02% for intra-day determination and −7.21 to 1.57% for inter-day determination, respectively. The corresponding precision (measured as RSD) ranged from 2.80 to 4.88% and 4.15 to 4.66%, respectively (Table 3). The developed LC–MS/MS method showed high sensitivity in determining MT with a LLOQ of 2 μg/kg. The stability evaluation showed that MT was stable in pig intestinal contents at −20°C for a week, at 26°C for 24 h, and after 3 freeze–thaw cycles (Supplementary Table S2).

**Table 3:** Precision and accuracy of the LC–MS/MS method for determining of MT in pig intestinal contents.

Nominal concentration (μg/mL)	Intra-day (*n* = 6)			Inter-day (*n* = 18)		
	Determined concentration (μg/L)	RSD (%)	RE (%)	Determined concentration (μg/L)	RSD (%)	RE (%)
2	2.02 ± 0.09	4.44	0.96	2.03 ± 0.08	4.15	1.57
	2.08 ± 0.08	3.90	4.02			
	1.99 ± 0.07	3.52	−0.27			
20	18.03 ± 0.73	4.07	−9.87	18.69 ± 0.87	4.63	−6.57
	18.59 ± 0.52	2.80	−7.03			
	19.43 ± 0.73	3.75	−2.83			
100	92.47 ± 4.51	4.88	−7.53	92.79 ± 4.33	4.66	−7.21
	90.75 ± 3.55	3.91	−9.25			
	95.15 ± 4.35	4.57	−4.85			

### 3.2 Kinetic behavior of MT in pig intestinal lumen

**Figure 3:**
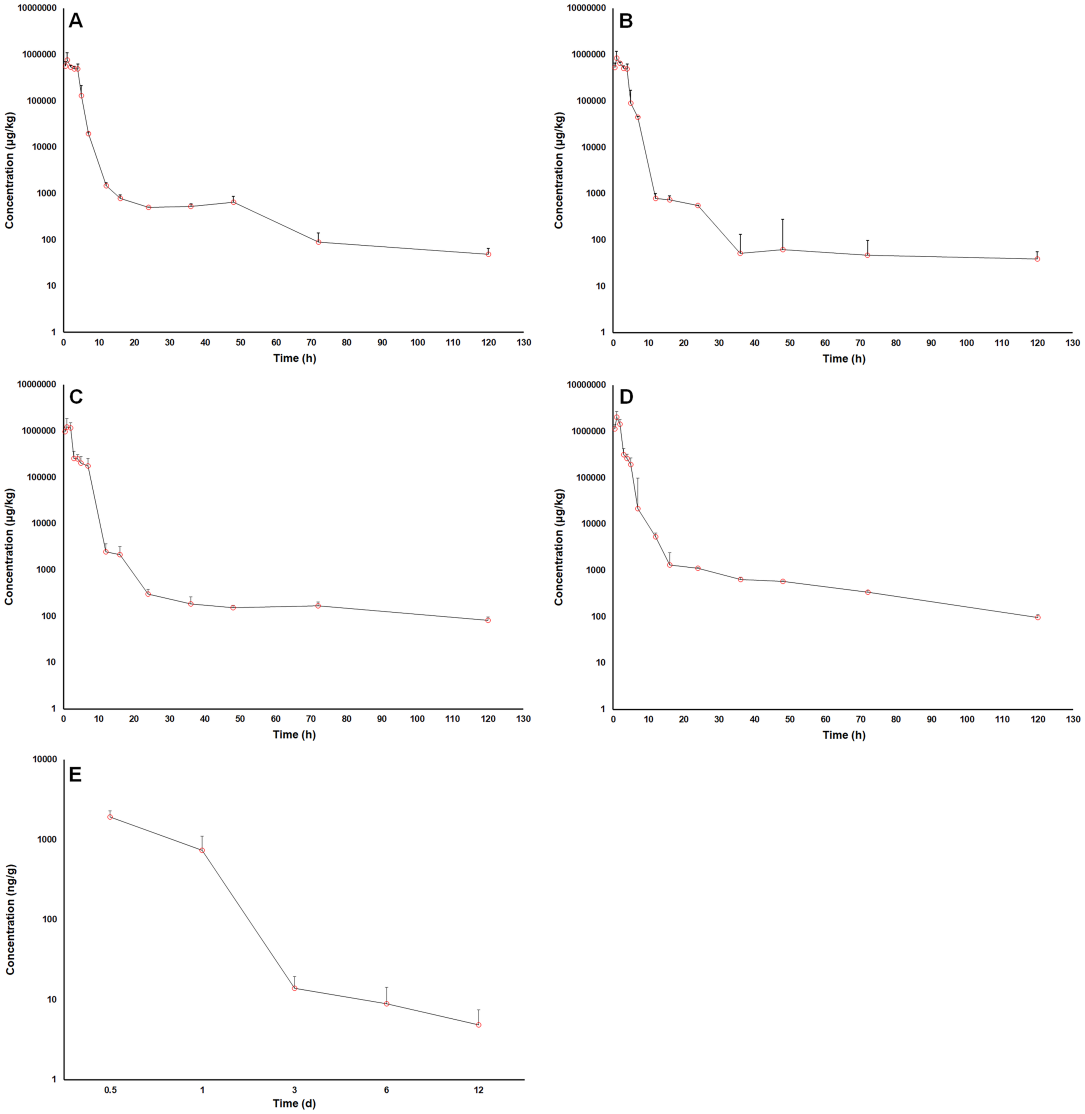
Log10 matrine concentrations in pig intestinal contents vs. time after oral administrations. **(A)** a single oral dose of matrine at 40 mg/kg, **(B)** a single oral dose of matrine at 40 mg/kg and amoxicillin at 40 mg/kg, **(C)** a single oral dose of matrine at 70 mg/kg, **(D)** a single oral dose of matrine at 70 mg/kg and amoxicillin at 70 mg/kg, **(E)** repeated oral doses of 50 mg/kg/day of matrine for consecutive 5 days.

The time courses of MT concentration in pig intestinal lumen after oral administrations were plotted in Figure 3. The main PK parameters were summarized in Table 4. After a single oral administration, the MT concentrations in pig intestinal lumen increased rapidly and reached their peaks within 2 h, and then decreased in a two-phase decay pattern (Figures 3A–D). That is, the MT concentrations in pig intestinal lumen decreased significantly at 2–24 h after administration, then the depletion of MT from pig intestinal lumen became very slow. At the last time point, 120 h after administration, the intestinal content samples collected from all pigs still had enough MT to be quantified. The kinetic behavior of MT in pig intestinal lumen after the combined administration of MT and AMO was similar to that after the administration of MT alone. No significant differences in C_max_, T_max_, AUC_0 → 120 h_, and Cl/F were found between the two groups (*p* > 0.05). After repeat oral administration, a relatively slow elimination of MT from pig intestinal lumen was observed (Figure 3E), and the mean t_1/2_ was 50.92 h. On day 12 after administration, the MT concentrations in all intestinal content samples were still above the LLOQ (2 μg/kg) of the analytical method.

**Table 4:** PK parameters (mean ± SD, *n* = 6) of MT in pig intestinal lumen after a single oral administration of MT alone and in combination with AMO.

Parameters	Unit	40 mg/kg		70 mg/kg	
		MT alone	MT in combination with AMO	MT alone	MT in combination with AMO
C_max_	μg/L	783916.7 ± 324337.1	888833.3 ± 309005.1	1,491,732 ± 437475.8	2,087,062 ± 1,578,140
T_max_	h	1.166667 ± 0.408248	1.5 ± 0.547723	1.416667 ± 0.66458	1 ± 0.547723
AUC_0 → 120 h_	h*μg/L	2,684,040 ± 359,993	2,848,776 ± 558384.4	4,030,908 ± 626463.8	4,519,267 ± 2,291,773
V/F	L/kg	0.47495 ± 0.092425	1.436533 ± 1.110726	1.750167 ± 0.508724^**^	0.757 ± 0.372851^**^
MRT	h	3.123183 ± 0.168564	3.1285 ± 0.316321	3.5674 ± 0.582906^*^	2.816417 ± 0.546953^*^
Cl/F	L/h/kg	0.0151 ± 0.001914	0.014417 ± 0.002384	0.017733 ± 0.002845	0.018817 ± 0.008131
λ_z_	1/h	0.0327 ± 0.006988	0.034117 ± 0.041753	0.010983 ± 0.003842^**^	0.025683 ± 0.005512^**^
t_1/2_	h	21.9341 ± 4.203353	68.82105 ± 49.43469	69.68718 ± 23.16356^**^	28.0228 ± 5.793749^**^

*represents significant difference (*P* < 0.05), and ** represents highly significant difference (*p* < 0.01). C_max_: maximum concentration; T_max_: time to maximum concentration; AUC_0 → 120 h_: area under the curve from time 0 to 120 h; V/F: apparent volume of distribution per kilogram of body weight; MRT: mean residence time; Cl/F: clearance per kilogram of body weight; λ_z_: terminal elimination rate constant; t_1/2_: terminal elimination half-life.

### 3.3 The PBPK model

#### 3.3.1 Model parameterization

**Figure 4:**
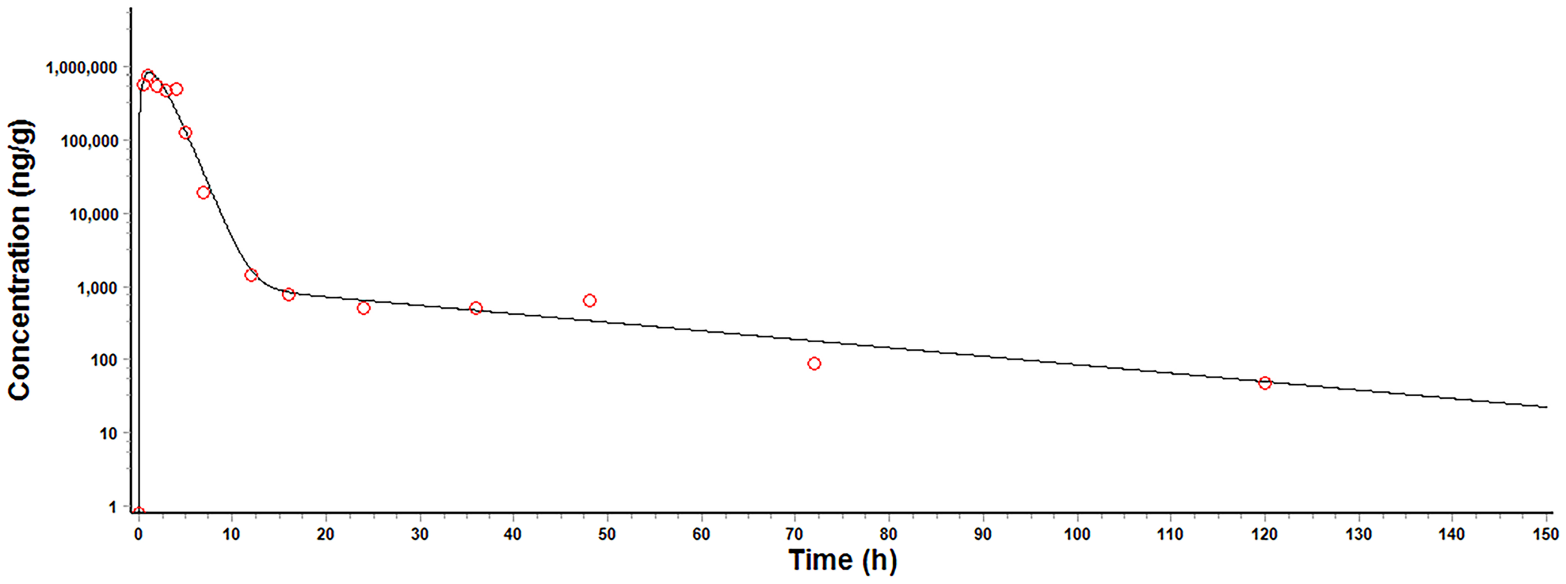
Final model simulation (solid line) against the observed (circles) matrine concentration in pig intestinal contents after a single oral dose of matrine at 40 mg/kg.

The plot of final model simulation against the observed MT concentrations in pig intestinal contents was presented in Figure 4, and the optimized parameters were summarized in Table 5. The model simulation closely matched the observed values. The volume of intestinal content (Vc_int_), k_st_, k_a_, k_e_, k_bi_, P_l_, and Cl_renal_ were within the acceptable range.

**Table 5:** Optimized parameters for PBPK model for MT in pig intestinal lumen.

Parameter	Symbol	Unit	Starting value	Optimized value	Data source
Cardiac output	Qcar	L/(h*kg)	4.944	4.944	(23)
Hematocrit	PCV	–	0.333	0.333	(23)
Blood flow of liver (% of Qcar)	Qc_l_	–	0.3	0.3	(23)
Blood flow of other organs (% of Qcar)	Qc_ot_	–	0.7	0.7	By calculation
Volume of intestinal content (% of BW)	Vc_int_	–	0.01	0.0136733	(23)
Volume of liver (% of BW)	Vc_l_	–	0.0294	0.0294	(23)
Volume of blood (% of BW)	Vc_b_	–	0.06	0.06	(23)
Volume of other organs (% of BW)	Vc_ot_	–	0.9006	0.8969267	By calculation
Rate of gastric emptying	k_st_	1/h	0.1	0.8544925	(21, 22)
Absorption rate constant	k_a_	1/h	0.3	0.8555538	By optimization
Bioavailability	F	-	0.171	0.7925891	(14)
Liver-to-blood partition coefficient	P_l_	–	5.5	2.936615	(15)
Other organs-to-blood partition coefficient	P_ot_	–	5	11.33514	By optimization
Fecal excretion rate constant	k_e_	1/h	0.01	0.007358172	By optimization
Biliary excretion rate constant	k_bi_	1/h	0.01	0.05834594	By optimization
Plasma protein binding	Pb	–	0.3	0.319	By optimization
Renal clearance	Cl_renal_	L/(h*kg)	1.182	0.2805897	(14)

The volume and blood flow of other organs were calculated as 1 minus the sum of the corresponding values for the remaining organs.

#### 3.3.2 Parameter sensitivity

All parameters were subjected to sensitivity analysis. However, only parameters with the absolute value of NSC greater than 0.1 were reported. The parameters, which increased the MT concentration in pig intestinal lumen when they were increased, were the volume of liver (Vc_l_), F, k_bi_ and P_l_. The tissue-to-blood partition coefficient of other organs (P_ot_) and k_st_ showed a dualistic effect on the model prediction. The remaining parameters (Vc_int_, k_a_, and Cl_renal_) had a negative effect on model prediction.

#### 3.3.3 Model predictive ability

**Figure 5:**
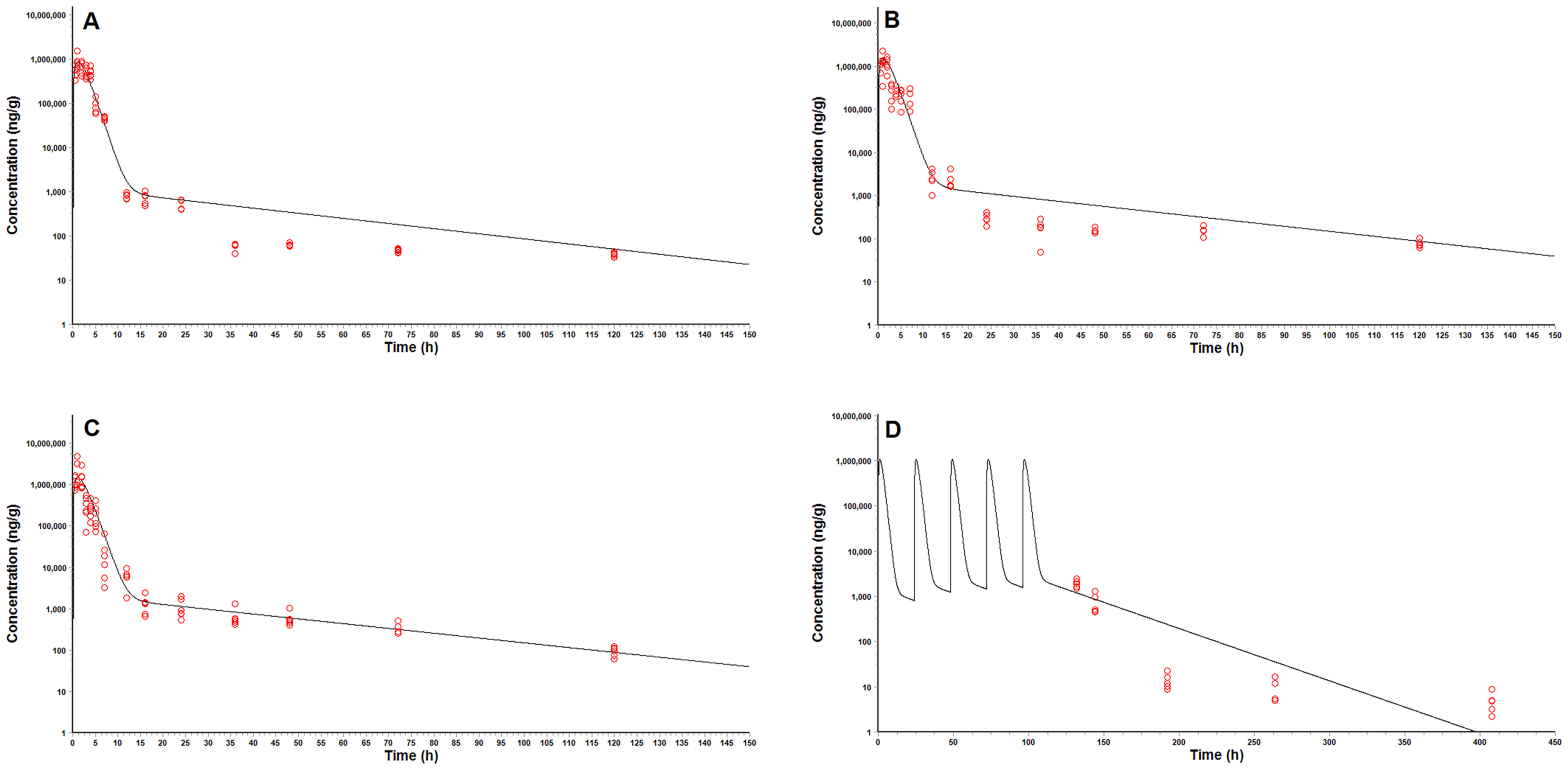
Comparison of model predicted (solid lines) and observed (circles) matrine concentrations in pig intestinal contents after oral administrations. **(A)** a single oral dose of matrine at 40 mg/kg and amoxicillin at 40 mg/kg, **(B)** a single oral dose of matrine at 70 mg/kg, **(C)** a single oral dose of matrine at 70 mg/kg and amoxicillin at 70 mg/kg, **(D)** repeated oral doses of 50 mg/kg/day of matrine for consecutive 5 days.

The predictive ability of our model was demonstrated by the acceptable agreement between the predicted and observed MT concentrations in pig intestinal contents after oral administrations (Figure 5). Linear regression analysis and Pearson correlation test also showed that the present model was generally acceptable because the correlation coefficients between the predicted and observed values were higher than 0.9409 and the model predictions were significantly correlation with the observed data (*p* < 0.05) (Table 6). One-sample t test showed that there were no significant differences between the predicted PK parameters and the corresponding values derived from experiment 1 (*p* > 0.05), which further validated the predictive ability of our model.

**Table 6:** The linear correlation and regression analysis between the predicted and observed MT concentrations in pig intestinal contents.

Dosage regimens	Slope (m)	Intercept (b)	Correlation coefficient (r)	Data source
MT in combination with AMO, 40 mg/kg	0.9543	14,525	0.9609**	Experiment 1
MT alone, 70 mg/kg	0.8065	−8,608	0.9611**	Experiment 1
MT in combination with AMO, 70 mg/kg	1.147	−58,717	0.9518**	Experiment 1
MT alone, 50 mg/kg once daily for 5 d	1.459	−149.4	0.9409*	Experiment 2

*represents significant difference (*P* < 0.05), and **represents highly significant difference (*P* < 0.01).

## 4 Discussion

Porcine colibacillosis has caused huge economic loss to pig industry. AMO is a *β*-lactam antibiotic that was once widely used to treat porcine colibacillosis. Unfortunately, AMO is becoming ineffective due to the increasing resistance to ETEC, which poses a challenge for the treatment of porcine colibacillosis. Recently, MT and total alkaloids extracted from the seeds of *Sophora alopecuroides* (TASA) have been found to restore the susceptibility of *E. coli* to a variety of antibiotics, including AMO (5–7), *in vitro*. Using AMO in combination with MT may be an effective strategy for addressing this challenge. In general, pig intestinal lumen has been considered to be the main site of infection, and the kinetic behavior of MT (administered alone or in combination with AMO) here is closely related to the therapeutic efficacy. In this study, for the first time, the kinetic behavior of MT in pig intestinal lumen was investigated after single oral administrations, and a minimal PBPK model was developed to predict its intestinal PK profiles. We believed our findings could be helpful to accurately evaluate the *in vivo* efficacy of MT and recommend an appropriate dosage regimen.

All concentration-time data were generated by experiments 1 and 2 based on a reliable LC–MS/MS method. The analytical performance of our method was well demonstrated by validation data as described above. The sample pretreatment procedure was optimized to achieve acceptable analytical specificity and accuracy. MT is a polar organic compound (weakly alkaline organic compound) that is easily soluble in polar solvents such as water, acetonitrile, and methanol (4). Acetonitrile was selected as an extraction solvent because of its high extraction efficiency and protein precipitation efficiency. The sample extracts were washed with ethyl acetate and then purified with X-Peony MCX SPE cartridge. During the washing step, most of the non-polar interferences was removed, while no analyte loss was observed. The X-Peony MCX SPE cartridge is specifically designed for the purification of weakly alkaline organic compounds from biological matrix. The volume and composition of the most critical washing solvent (ammonium hydroxide solution) were optimized to remove co-extracted polar interferences while retaining the analyte. Satisfactory purification results and extraction efficiency were obtained after the intestinal content samples were applied to an X-Peony MCX SPE cartridge (300 mg, 6 mL) and washed with 3 mL of ammonium hydroxide solution (0.25%, v/v). Significant matrix suppression effect was observed in the analysis of intestinal content samples. A matrix-matched calibration curve was therefore used to compensate for matrix effect and to ensure accurate quantification. The curve showed good linearity over the concentration range of 1 to 100 μg/L (r > 0.999). The incurred samples containing excessive analyte concentrations (> 100 μg/kg) were diluted with blank sample extracts prior to analysis so that they fell within the linear range of the matrix matching standard curve. The LLOQ of our LC–MS/MS method was 2 μg/kg. The high analytical sensitivity ensured the acquisition of as much concentration-time data as possible to truly reflect the kinetic behavior of MT in pig intestinal lumen.

After a single oral administration, the MT concentrations in pig intestinal lumen increased rapidly and reached their peaks within 2 h. Gastric emptying is the main cause of increasing MT concentration in pig intestinal lumen. Many factors such as the volume and composition of gastric contents can affect the rate of gastric emptying (26). In Experiment 1, all pigs were fasted for 12 h prior to administration, then provided with a small amount of medicated feed (approximately 100 g), followed by ad libitum access to food and water. This was done to ensure that MT was ingested as much as possible, but it also led to two consequences. One was that sufficient intestinal contents could not be collected at the first sampling point (0.25 h after administration). As a result, the data from the first sampling point were lost, and incomplete “absorption phases” (the changes in MT concentration within 2 h after administration) were presented in Figures 3A–D. Nonetheless, based on the available data from subsequent time points (e.g., 0.5 h and beyond), the overall trend of the absorption phase (within 2 h post-administration) remains discernible (Figures 3A–D). The other was overfeeding of these hungry pigs accelerated gastric emptying, resulting in a rapid increase in MT concentration in pig intestinal lumen. A two-phase decrease in MT concentrations in pig intestinal lumen was observed 2 h after administration. This suggested that the change in MT concentration in pig intestinal lumen might not only be caused by absorption into the blood stream and fecal excretion. There might also be other rate processes that affected the MT concentration in intestinal lumen. One explanation was that biliary excretion might be involved in the transport of MT between intestinal lumen and the body. Biliary excretion would increase MT concentration in intestinal lumen, while absorption and fecal excretion would decrease MT concentration in intestinal lumen. At 2–24 h after administration, the rate of biliary excretion appeared to be much lower than that of absorption and fecal excretion. The MT concentration was therefore decreased significantly during this period. With the passage of time, the rate of biliary excretion gradually increased, while the rate of absorption and fecal excretion gradually decreased. As a result, the depletion of MT from pig intestinal lumen became very slow.

Similar kinetic behaviors were observed when MT was administered alone and in combination with AMO (especially at low dose), suggesting that AMO did not affect the rate of gastric emptying, nor did it alter the rate processes of MT in pig intestinal lumen. However, when the dose was very high, the effect of AMO on the kinetic behavior of MT might manifest itself, as the competition between the two drugs for digestive fluids would significantly affect their absorption and fecal excretion.

Previous *in vitro* experiment showed that MT at concentrations of 300–500 μg/mL reversed the resistance of *E. coli* to ciprofloxacin (6). When the pigs received a single oral dose of MT at 40 mg/kg, the mean C_max_ was 1.57–2.61 times higher than the effective concentrations measured *in vitro*, but the time that MT concentrations in pig intestinal lumen exceeded 300 μg/g did not exceed 2 h. Repeated dosing is required to achieve a sufficient exposure.

A minimal PBPK model for MT in pig intestinal lumen was developed and applied to pharmacodynamic evaluation and dosage regimen design. The model was simplified as much as possible. Only the core compartments were included in the model structure. The distribution of MT in these compartments was described as flow-limited. For most small-molecule polar water-soluble compounds that possess sufficient lipid solubility, passive diffusion acts as the dominant transmembrane transport pathway, a pathway that inherently follows a first-order kinetic process (27). Consequently, all rate processes involved were considered to be kinetically first-order. PK study in rat showed that MT did not undergo first-pass metabolism after oral administration (14). The metabolism of MT was therefore excluded from the model. MT might accumulate in specific tissues because of its relatively high apparent volume of distribution (14, 16) and tissue-to-blood partition coefficients (15). However, the rate processes associated with the tissue binding of MT were omitted due to the limited PK data available. Such simplifications might cause the prediction bias (Figures 5A,B, 24–120 h after administration; Figure 5D, 192–408 h after administration). Nevertheless, our model still gave an accurate prediction of MT concentrations in pig intestinal lumen at most time points. According to our model predictions, a dosage regimen of 70 mg/kg every 8 h was recommended to ensure a sufficient exposure (the predicted C_max_ was 2.15–3.59 times higher than the effective concentrations measured *in vitro* (300 μg/g), and the percentage of a 24-h time period that predicted MT concentrations in pig intestinal lumen exceeded the effective concentrations measured *in vitro* was more than 55.75%). It should be noted that the concentrations of MT in the existing *in vitro* experiments (> 300 μg/mL) are too high compared with the concentrations that can be achieved at the site of infection after administration. More *in vitro* experiments are needed to elucidate the dose–response relationship of MT in drug-resistant ETEC, especially at low concentrations.

Experiment 2 generated a set of concentration-time data, which was used to evaluate the performance of our PBPK model. Notably, the pigs in experiment 2 differed from those in experiment 1 in terms of BW and breed. BW and breed may be the factors that affect the intestinal kinetics of MT. The effect of BW on the intestinal kinetics of MT was evaluated by sensitivity analysis. The results showed that the absolute value of NSC for BW was much less than 0.1, indicating that BW had little to no effect on the intestinal kinetics of MT. Regarding the effect derived from breed, a quantitative evaluation cannot be performed temporarily due to the lack of comparative physiological parameters (such as the k_st_, Vc_i_, Qcar, and Qc_i_) of ternary crossbred pigs (Landrace × Yorkshire × Duroc) and binary crossbred pigs (Landrace × Yorkshire). These parameters are vital to the PBPK model, as they reflect interspecies differences, and their absence precludes quantifying the specific impact of breed on the disposition of MT in the body. Nevertheless, the effect of breed on the intestinal kinetics of MT can be indirectly evaluated based on the consistency between the model predictions and the observed values from experiment 2. Despite differences from experiment 1 in terms of BW and breed, our PBPK model remained capable of accurately predicting the intestinal kinetics of MT in pigs (Figure 5D). This not only confirms that our understanding of the intestinal kinetic behaviors of MT is fundamentally accurate, but also demonstrates the robustness of our PBPK model in the face of changes to non-core experimental conditions (differences in BW and breed). In other words, neither BW nor breed is likely to significantly alter the intestinal kinetic behavior of MT in pigs.

Several limitations existed in our study. The first limitation was the loss of samples at the first sampling point, which resulted from fasting the animals before drug administration. Finally, the temperature (25.8 ± 3.2°C) and humidity (76.5–87.1%) in our animal housing facilities were slightly outside the commonly recommended ranges for this category of pigs [typically 13–27 C and 50–80%, as referenced in GB/T 17824.3–2008 (28)]. This deviation was due to unexpected seasonal climate fluctuations during the experimental period, despite our efforts to maintain controlled environmental conditions using ventilation and humidification systems. Nevertheless, no observable clinical signs of discomfort or stress (e.g., altered feed intake, abnormal behavior) were detected in the pigs. Theoretically, the occurrence of such signs could alter the kinetic behavior of MT in the pig intestinal lumen.

## 5 Conclusion

In summary, the kinetic behavior of MT in pig intestinal lumen was investigated and a minimal PBPK model for MT in pig intestinal lumen was developed. After oral administrations, the MT concentrations in pig intestinal lumen increased rapidly and reached their peaks within 2 h, then decreased in a two-phase decay pattern. The co-administered AMO did not alter the kinetic behavior of MT in pig intestinal lumen. Our PBPK model gave an accurate prediction of MT concentrations in pig intestinal lumen at most time points. According to the model predictions, a dosage regimen of 70 mg/kg every 8 h was recommended to ensure a sufficient exposure.

## Data Availability

The original contributions presented in the study are included in the article/Supplementary material, further inquiries can be directed to the corresponding author.
