# Supplementary material for: The kinetic behavior of matrine in pig intestinal lumen after oral administration and its physiologically based pharmacokinetic modeling
Source: Front Vet Sci. 2025 Oct 14;12:1620161. doi: 10.3389/fvets.2025.1620161 (PMC12558759; doi:10.3389/fvets.2025.1620161)
Supplement: Supplementary file 1 [file Table_1.DOCX]

Supplementary Material

# Supplementary Tables

Table S1 The optimized MS/MS parameters for MT.

| Qualitative ions (m/z) | Quantification ions (m/z) | Dwell time (s) | Cone voltage (V) | Collision energy (V) | Capillary voltage (Kv) | Source temperature (°C) |
| --- | --- | --- | --- | --- | --- | --- |
| 249.1 → 176.0 | 249.1 → 148.0 | 0.1 | 100 | 35 | 3.0 | 350 |
| 249.1 → 148.0 |  | 0.1 | 100 | 35 |  |  |

Table S2 Storage stability of MT in pig intestinal contents.

| Nominal concentration (μg/mL) | Condition | Ratio of determined and nominal concentration (%, n = 6) | RSD (%) |
| --- | --- | --- | --- |
| 2 | –20 °C, a week | 102.67±4.38 | 4.27 |
|  | room temperature (26 °C), 24h | 106.66±3.90 | 3.66 |
|  | 3 freeze-thaw cycles | 95.95±8.71 | 9.07 |
| 20 | –20 °C, a week | 93.63±2.80 | 2.99 |
|  | room temperature (26 °C), 24h | 99.18±1.75 | 1.77 |
|  | 3 freeze-thaw cycles | 101.30±8.90 | 8.79 |
| 100 | –20 °C, a week | 94.84±4.02 | 4.23 |
|  | room temperature (26 °C), 24h | 96.47±1.28 | 1.33 |
|  | 3 freeze-thaw cycles | 98.33±2.89 | 2.94 |
